# Mapping Compulsivity in the DSM-5 Obsessive Compulsive and Related Disorders: Cognitive Domains, Neural Circuitry, and Treatment

**DOI:** 10.1093/ijnp/pyx088

**Published:** 2017-09-23

**Authors:** Naomi A Fineberg, Annemieke M Apergis-Schoute, Matilde M Vaghi, Paula Banca, Claire M Gillan, Valerie Voon, Samuel R Chamberlain, Eduardo Cinosi, Jemma Reid, Sonia Shahper, Edward T Bullmore, Barbara J Sahakian, Trevor W Robbins

**Affiliations:** 1 Hertfordshire Partnership University NHS Foundation Trust, Welwyn Garden City, Hertfordshire, United Kingdom; 2 University of Hertfordshire, Department of Postgraduate Medicine, College Lane Hatfield, United Kingdom; 3 Department of Psychiatry, School of Clinical Medicine, University of Cambridge, Addenbrooke’s Hospital, Cambridge, United Kingdom; 4 Behavioral and Clinical Neurosciences Institute, University of Cambridge, Cambridge, United Kingdom; 5 Department of Psychology, University of Cambridge, Cambridge, United Kingdom; 6 Cambridge and Peterborough NHS Foundation Trust, Cambridge, United Kingdom; 7 School of Psychology, Trinity College Dublin, Dublin, Ireland; 8 Global Brain Health Institute, Trinity College Dublin, Dublin, Ireland

**Keywords:** cognitive domains, neural circuitry, treatment

## Abstract

Compulsions are repetitive, stereotyped thoughts and behaviors designed to reduce harm. Growing evidence suggests that the neurocognitive mechanisms mediating behavioral inhibition (motor inhibition, cognitive inflexibility) reversal learning and habit formation (shift from goal-directed to habitual responding) contribute toward compulsive activity in a broad range of disorders. In obsessive compulsive disorder, distributed network perturbation appears focused around the prefrontal cortex, caudate, putamen, and associated neuro-circuitry. Obsessive compulsive disorder-related attentional set-shifting deficits correlated with reduced resting state functional connectivity between the dorsal caudate and the ventrolateral prefrontal cortex on neuroimaging. In contrast, experimental provocation of obsessive compulsive disorder symptoms reduced neural activation in brain regions implicated in goal-directed behavioral control (ventromedial prefrontal cortex, caudate) with concordant increased activation in regions implicated in habit learning (presupplementary motor area, putamen). The ventromedial prefrontal cortex plays a multifaceted role, integrating affective evaluative processes, flexible behavior, and fear learning. Findings from a neuroimaging study of Pavlovian fear reversal, in which obsessive compulsive disorder patients failed to flexibly update fear responses despite normal initial fear conditioning, suggest there is an absence of ventromedial prefrontal cortex safety signaling in obsessive compulsive disorder, which potentially undermines explicit contingency knowledge and may help to explain the link between cognitive inflexibility, fear, and anxiety processing in compulsive disorders such as obsessive compulsive disorder.

## Introduction

Compulsions are stereotyped behaviors, performed according to rigid rules and designed to reduce or avoid unpleasant consequences (Chamberlain et al., 2009). The newly created DSM-5 Obsessive Compulsive and Related Disorders (OCRDs) (APA 2013) are defined by the presence of compulsions. However, compulsive behaviors are observed in many other psychiatric disorders, particularly those involving deficient impulse control. For example, much of the behavior associated with disorders of eating, substance addiction, and “behavioral addiction,” such as pathological gambling or problematic internet usage (Ioannidis et al., 2016), is theorized to shift over the course of time from reward-driven impulsive (rapid, reckless) to compulsive activity (Everitt and Robbins, 2005; Robbins et al., 2012). These disorders share a profound experience of “lack of control,” thought to derive from the dysfunctional inhibition of thoughts and behaviors naturally prone to excess, for example, grooming, eating, purging, gambling, and checking. As poorly understood lifespan disorders, they are difficult to treat and responsible for considerable psychiatric (depression, suicide) and somatic morbidity and cost to the individual and society as a whole (Hollander et al., 2016).

Regrettably, the development of new treatments in psychiatry is slowing, related, at least in part, to difficulties translating positive results from experiments using nonhuman illness models to the clinical setting. These difficulties align with growing concern about the scientific utility of the existing diagnostic systems (ICD-10, World Health Organisation 1992, DSM-5, APA 2013) that tend to define psychiatric disorders according to symptoms and syndromes and give less prominence to neuropsychological substrates. It is thought likely that the considerable biological heterogeneity that exists within the current taxonomy is hampering identification of the underpinning mechanisms that may serve as new therapeutic targets. In response, the EU Roadmap for Mental Health Research in Europe (Haro et al., 2014) and the US National Institute of Mental Health, Research Domain Criteria (https://www.nimh.nih.gov/research-priorities/rdoc; Insel et al., 2010) have called for new ways of classifying psychopathology to better support treatment development based on dimensions of observable behavior with established biological validity, irrespective of diagnosis. Identifying the pathophysiological mechanisms underpinning compulsivity as a trans-diagnostic, neuropsychological domain would therefore be expected to advance the search for new treatment targets and support innovation in developing evidence-based treatments (Fineberg et al 2013a).

Many different compulsive disorders are found clustered within the same individual (comorbidity) or within the families of affected individuals, implying that vulnerability to these disorders is mediated via shared pathophysiological mechanisms (Fineberg et al., 2014). The investigation of endophenotypes (intermediate phenotypes) that lie closer than do the expressed behaviors (phenotypes) to the genetic and environmental origins of compulsive disorders (Gottesman and Gould, 2003; Chamberlain and Menzies, 2009), such as changes in cognitive performance, or structural and functional brain imaging abnormalities, is expected to provide a clearer understanding of the biological processes underpinning these disorders.

Based on emerging data from the neurosciences, this narrative review, which was first delivered as a plenary lecture at the 2016 Annual Congress of the International College of Neuropsychopharmacology (Fineberg et al., 2016), appraises the results of a decade of research by the authors dedicated to exploring the neuropsychological underpinnings of the OCRDs, as examples of compulsive disorders, from the perspective of diagnosis, evidence-based treatments, candidate neuro-psychological endophenotypes, and associated neural circuitry. The research builds upon previous and ongoing research by other groups and, as it has progressed, has generated testable models of compulsivity as a biologically relevant trans-diagnostic domain that could be expected to advance diagnostic classification and identify new avenues for treatment, including novel psychological, pharmacological, and somatic treatment targets for these disabling and intransigent disorders.

### The Obsessive-Compulsive and Related Disorders

The DSM-5 OCRD cluster, comprising obsessive–compulsive disorder (OCD), body dysmorphic disorder (BDD), hoarding disorder, hair-pulling disorder, and skin-picking disorder, represents some of the most costly, functionally disabling, and treatment-resistant brain disorders. By gathering together diagnoses previously listed in the DSM-IV under Anxiety Disorders, Somatoform Disorders, and Impulse-Control Disorders Not Elsewhere Classified, this new classification aims to advance the scientific study of the disorders as well as to improve their clinical recognition and management. The disorders commonly occur together and yet are surprisingly poorly recognized, as individuals are often not forthcoming about their symptoms (e.g., due to a sense of shame, or lack of knowledge that these problems constitute recognized mental disorders). As a result, there is usually a considerable time-lag, in the case of OCD, amounting to approximately 15 years, before the correct diagnosis is made and the correct treatments initiated. The duration of untreated illness represents one of the principal factors determining clinical and health outcomes (Dell’Osso et al., 2013), emphasizing the importance of early detection, especially for child and adolescent onset OCD (Fineberg et al., 2013a). It is to be hoped that by introducing this new classification, clinicians would be more likely to enquire about and detect the other disorders. It is fully expected that the forthcoming ICD revision will adopt a similar approach and may even include additional new diagnoses among the OCRD grouping, such as olfactory reference syndrome (Marras et al., 2016).

OCRDs are generally thought to be highly heritable (hoarding disorder, Iervolino et al., 2009; OCD, Nestadt et al., 2010; BDD, Monzani et al., 2012) lifespan disorders (reviewed in Steketee, 2011; APA 2013), though episodic forms of OCD, hair pulling, and skin picking are seen. They are characterized by the irresistible urge to perform distressing and time-consuming compulsive acts. Of these disorders, OCD has been subject to most study and is arguably the most well understood. OCD affects approximately 3% of the general population, though only a fraction of affected individuals present for treatment. Of considerable interest, subthreshold OCD is found to be extremely common and such cases share a similar age of onset and symptom trajectory as OCD, suggesting a natural continuum of compulsive behavior exists affecting as many as 20% of the general population (Fineberg et al., 2013b).

Patients with OCD show difficulty in flexibly shifting attentional focus away from distressing intrusive, perseverative thoughts (obsessions) and behaviors (compulsions) (Fineberg et al., 2010, 2014). Washing, checking, ordering, and arranging compulsions are extremely common. Whereas traditional learning-based psychological models of OCD posit harm avoidance as the major reinforcer of compulsive behaviors, some symptoms of OCD, especially those concerned with ordering and arranging to achieve symmetry, appear to reflect a need to make the environment “feel right.” Growing evidence suggests these symptoms represent a separate OCD subgroup, in which compulsions are driven by the urge to avoid an unpleasant “not just right feeling.” The “not just right feeling” has been found to be associated with an earlier age of OCD onset and the presence of sensory processing difficulties (Hellriegel et al., 2016), implicating the involvement of neuro-developmental mechanisms akin to autism spectrum disorder (ASD) in its etiology.

Hoarding disorder is a separate, poorly understood, and highly treatment refractory OCRD that involves the compulsive acquisition of new items and difficulty discarding owned items. These hoarding behaviors can also be viewed as an expression of the need to make the environment “feel right.” Typical responses when asking individuals what they would think/feel if we threw out a “treasured item,” which may be an old sweet paper, bus ticket, etc. are “it feels like I am missing part of me, it does not feel right” (Frost and Steketee, 2010). Hoarding compulsions are also commonly found in patients with OCD as well as those with neurodevelopmental disorders such as ASD. In young people with OCD, hoarding is associated with prominent executive function deficits (Park et al., 2016). Hair-pulling disorder and skin-picking disorder, on the other hand, are defined by more obviously disinhibited behavior, in the form of repetitive, body-focused grooming habits that can be considered as either predominantly impulsive or compulsive, depending on the nature of the symptoms expressed (Chamberlain et al., 2007).

Other phenotypic signs of an altered neurodevelopmental trajectory are also commonly observed in patients with OCRDs, such as traits or symptoms of tic disorder, ASD, and attention deficit hyperactivity disorder. These comorbid traits and diagnoses appear to cluster in the same patient or within their family members, hinting that shared, heritable neuro-behavioral mechanisms contribute to the expression of many compulsive disorders (de Vries et al., 2016; Wikramanayake et al., 2017). Cases of tic-related OCD tend to have a male predominance (similar to ASD), an earlier age of onset, and a higher proportion of OCD symptoms related to symmetry, “not just-right experiences,” and forbidden thoughts compared with non-tic-related OCD (Prado et al., 2008). Additionally, tic-related OCD shows a more favorable response to adjunctive treatment with dopamine antagonist drugs (Bloch et al., 2006). In response to the emerging evidence, the DSM-5 has highlighted the presence of tic as the first neuro-behavioral specifier of a clinically relevant OCD subtype. Studies in patients with Tourette’s syndrome indicate a complex genetic relationship exists between tic disorder, OCD, and attention deficit hyperactivity disorder. One such recent study (Darrow et al., 2016) identified two independent, heritable, symptom-based factors (one involving the urge to attain symmetry, the other involving behavioral disinhibition) as possible trans-diagnostic phenotypes of compulsive behavior.

### Evidence-Based Treatment of OCD and Related Disorders

**Figure 1.**
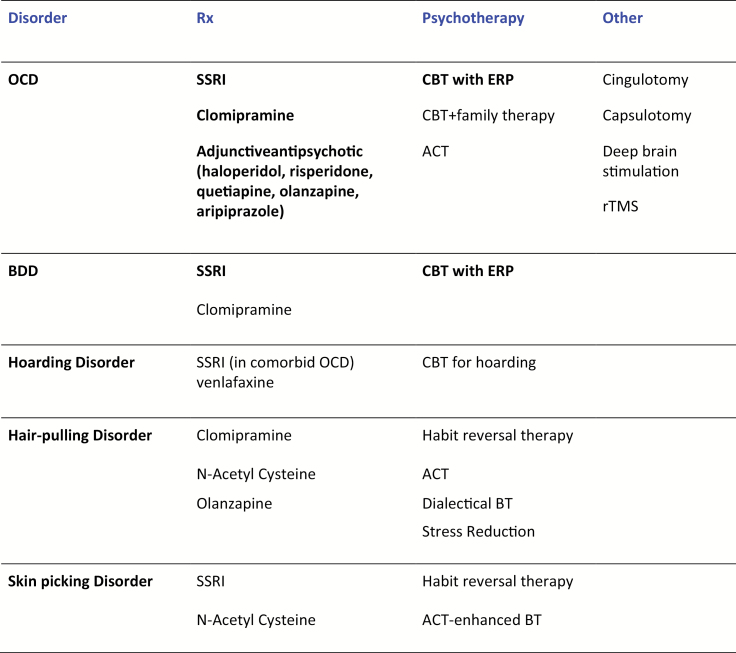
Evidence-based treatments for OCRDS. Adapted from Grant J, Chamberlain S, Odlaug B, Clinical Guide to OCRDs, Oxford, 2014a. Treatments with robust evidence of efficacy derived from randomized controlled trials of fair comparison are highlighted in bold black type. ACT,acceptance and commitment therapy; BDD, body dysmorphic disorder; BT,behavior therapy; CBT,cognitive behavior therapy; ERP,exposure and response prevention; OCD, obsessive compulsive disorder; rTMS,repetitive transcranial magnetic stimulation; Rx, Medication; SSRI,selective serotonin reuptake inhibitor.

Figure 1 summarizes the evidence-based treatment of the OCRDs based on a systematic review (Grant et al., 2014a). Apart from some studies of OCD and BDD, the pharmacotherapies were almost exclusively tested in small un-replicated trials, and the psychotherapies were not rigorously tested against a matched control of fair comparison. Randomized controlled treatment trials of adequate size and power to enable the detection of predictive outcome markers are urgently needed to drive forward the clinical management of these disorders on an individualized basis (Sachdev et al., 2017).

OCD typically responds to pharmacological treatment with serotonin reuptake inhibitors (SRIs; clomipramine and selective SRIs, SSRIs) according to a dose-response relationship (higher doses needed for better clinical response) or to SSRIs combined with antipsychotic agents, and to cognitive behavior therapy (CBT) involving exposure and response prevention (ERP) (Fineberg et al., 2015). Antipsychotics represent first-line treatment for Tourette’s syndrome and adjunctive antipsychotic may be preferentially effective in OCD with comorbid tics (Bloch et al., 2006). BDD has been studied less intensively than OCD but also shows a similar treatment response (Veale et al., 2014; Rashid et al., 2015; Phillips et al., 2016), though it remains less clear as to whether higher SSRI dosages and adjunctive antipsychotic are of value (reviewed in Reghunandanan et al., 2015c). The compulsions associated with ASD also respond to SSRI, though the increased risk of SSRI-induced adverse effects in the autistic population, such as behavioral activation and agitation, warrant care in dosage titration and subject selection (Kolevzon et al., 2006). Hoarding behavior has been mainly studied in the context of comorbid OCD and may respond to SSRI or venlafaxine (Saxena and Sumner, 2014), but as yet no effective pharmacological treatment has been established for primary hoarding disorder. CBT, even when delivered intensively over long periods, has so far been found to produce only limited improvement in hoarding behavior (Uhm et al., 2016). In hair-pulling disorder the data supporting the efficacy of SSRI and clomipramine are also not strong. Unlike OCD, but similar to impulse control disorders, SSRIs appeared to have a rapid onset of effect which was not sustained over time (Rothbart et al., 2013). Habit reversal therapy, rather than ERP, has emerged as the psychological therapy of choice (McGuire et al., 2014). Other data from single randomized controlled trials in hair-pulling disorder suggest that olanzapine (an antipsychotic agent) (Van Ameringen et al., 2010) and n-acetyl cysteine (an amino acid compound) (Grant et al., 2009) could be effective. Naltrexone, an opiate antagonist, produced substantial benefits in a small open-label study of children with hair pulling disorder (De Sousa, 2008), but the drug was not effective in a double-blind placebo-controlled study. However, those in this study with a family history of addiction showed a greater (but not statistically significant) decrease in the urge to hair-pull (Grant et al., 2014b). Skin picking disorder has been barely studied to date, but as with hair-pulling disorder, shows some response to SSRI and n-acetyl cysteine (reviewed in Reghunandanan et al., 2015c).

**Figure 2.**
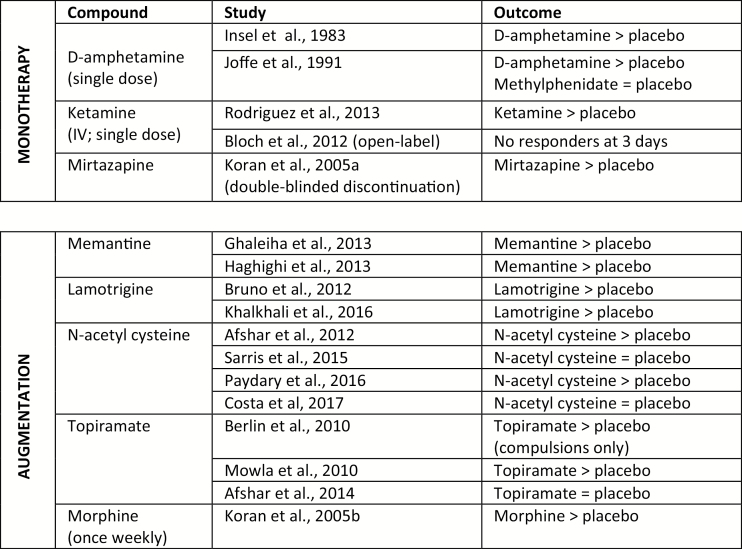
SSRI-Resistant OCD: Small sized randomized controlled trials showing efficacy vs. placebo.

Approximately 40% of OCD patients fail to respond to standard forms of therapy (Fineberg et al., 2015). Of great interest, a wide range of pharmacological compounds have been tested in treatment-resistant OCD and some have been found to be effective in small-sized trials, implicating a multiplicity of potential treatment targets and mechanisms (see Figure 2)

Thus, notwithstanding the limitations of the study data, some compulsive disorders (e.g., OCD, BDD) exhibit a strikingly similar treatment response to SRIs, suggesting that the underpinning neural mechanisms may overlap significantly, and possibly also with those relating to anxiety and affective disorders. In contrast, hair pulling and skin picking disorders, which are also characterized by prominent impulse control and addictive symptomatology, respond better to treatment with drugs acting on dopamine, glutamate, opioid, and noradrenergic systems, that is, potentially more like impulse-control disorders or even behavioral addictions (e.g. Grant et al., 2014b), whereas those with SRI-resistant OCD respond to similar agents in combination with SSRI, and OCD with prominent motor symptoms (tics, habits) may respond preferentially to adjunctive dopamine antagonists, known to modulate the cortico-striatal motor circuitry involved in Tourette’s syndrome and animal models of excessive habit behavior (Fineberg et al., 2014; Furlong et al., 2014).

The pharmacological treatment response may be of particular value for parsing psychiatric disorders and defining the boundaries of diagnostic groups, as it depends on underpinning biological mechanisms. As the treatment trial data for the OCRDs accrues, it is possible that for some disorders more convincing similarities will be found with disorders classified elsewhere in the DSM, such as the behavioral addictions (e.g., pathological gambling), impulse control disorders (e.g., intermittent explosive disorder), or even neurodevelopmental disorders (e.g. ASD), challenging their classification within the OCRDs grouping. Alternatively, by taking a dimensional (impulsive-compulsive-habit) approach to the psychopharmacology of the OCRDs, the emerging evidence may instead be interpreted to support the inclusion of some of these other disorders into an expanded OCRDs grouping (Sachdev et al., 2017).

### Neuropsychological Endophenotypes

Psychiatric symptoms and cognitive deficits can be conceptualized as disordered structure, connectivity, and function in large-scale neural networks. A series of evolutionarily well-conserved, parallel, cortico-striato-thalamo-cortical (CSTC) circuits are believed to underpin the expression of compulsive behaviors (Alexander et al., 1986; Cummings et al., 1993; Groenewegen and Uylings 2000). These circuits include direct (positive feedback) and indirect (negative feedback) pathways, projecting from specific cortical areas to the corresponding subregions of the striatum and thalamus with recurrent projections to the cortex. They are involved in diverse computational activities, including reward processing, action selection, habit formation, and motor control (Arnsten et al., 2011; Robbins et al., 2012). They play an important role in recognizing behaviorally significant stimuli (and in error detection) and in regulating goal-directed responses (Lovinger, 2010) and may therefore be particularly important for OCRDs. The anatomical overlap and functional interplay between these circuits may explain why compulsive behavior occurs in so many psychiatric syndromes.

### Historical Perspectives

Early indications that the compulsive behaviors seen in OCD and other compulsive disorders may be mediated by CSTC circuits (reviewed in Reghunandanan et al., 2015a) included research showing an association between postencephalitis Parkinsonian, obsessive-compulsive symptoms and striatal lesions (Cheyette and Cummings, 1995). OCD symptoms were also found to occur in a range of other neurological disorders involving striatal pathology, including Tourette’s syndrome, Sydenham’s chorea, Huntington’s disorder, and Parkinson’s disorder (Pitman et al., 1987; Rapoport, 1989; Stein et al., 1994). OCD patients have subsequently been found to demonstrate abnormalities in a broad series of measures used in neuropsychiatric (e.g., neurological soft signs, olfactory identification, evoked potentials, intra-cortical inhibition) and neuropsychological (e.g., executive function) research (Stein et al., 1994; Purcell et al., 1998a). These abnormalities have consistently implicated CSTC dysfunction and impaired control of the inhibition of thoughts and behaviors (reviewed in Morein-Zamir et al., 2010). Some evidence has suggested that they are relatively specific to disorders characterized by compulsive behaviors (Purcell et al., 1998b; Phillips et al., 2010).

Advances in brain imaging have provided persuasive neuroanatomical data for OCD (Rauch and Baxter, 1998; Graybiel and Rauch, 2000; Whiteside et al., 2004; Mataix-Cols and van den Heuvel, 2006; Menzies et al., 2007; Milad and Rauch, 2012) as well as Tourette’s syndrome (Groenewegen et al., 2003), trichotillomania (Chamberlain et al., 2009), impulse control disorders in Parkinson’s disease (van den Heuvel et al., 2010), and addictive disorders (Everitt and Robbins, 2005). Functional imaging in OCD has demonstrated increased activity in CSTC circuits connecting the orbitofrontal cortex, cingulate cortex, and striatum, both at rest and especially during exposure to feared stimuli (reviewed in Reghunandanan et al., 2015a). Somewhat different circuits may be involved in mediating different OCD symptom clusters such as hoarding (Saxena et al., 2004; Mataix-Cols et al., 2005). The use of sophisticated cognitive and affective paradigms has generated new heuristics regarding the role of these circuits (Fitzgerald et al., 2004; Remijnse et al., 2006); for example, during implicit learning, OCD subjects failed to show an expected increase in striatal activity and instead activated temporal cortex regions (Rauch et al., 2001). The observation that some behavioral challenges, such as exposure to OCD cues, induce over-activation of the orbitofrontal cortex on functional imaging and others induce underactivation (e.g. Chamberlain et al., 2008) may be explained by functional segregation within the orbitofrontal cortex.

Pediatric imaging research has also supported the involvement of CTSC circuits in OCD (reviewed in Reghunandanan et al., 2015a) and potentially offers the promise of being able to determine the evolution of brain abnormalities over time (Rosenberg and Keshavan, 1998, Rosenberg et al., 2011). Abnormal structure or function in other brain regions such as the temporal lobe structures involved in memory and fear processing (Hugo et al., 1999; Zungu-Dirwayi, 1999, Szeszko et al., 1999) and the supra marginal gyrus and the parietal lobe involved in the initiation and flexible control of instrumental behavior (Chamberlain et al., 2008; Meunier et al., 2012) have less commonly been found in OCD. However, recent meta- and mega-analyses of structural imaging data collected from research sites worldwide found distinct patterns of subcortical abnormalities in pediatric and adult OCD patients. The hippocampus as well as the pallidum seemed to be of importance in adult OCD, whereas the thalamus was involved in pediatric OCD (Boedhoe et al., 2017).

Both successful SRI pharmacotherapy and behavioral therapy have been shown in OCD to normalize activity in CSTC circuits (Baxter et al., 1992). Baseline structure or activity may differentially predict response to pharmacotherapy and psychotherapy (Brody et al., 1998; Hoexter et al., 2013) suggesting that different treatment modalities exert their clinical effects via different neuro-mechanisms (Reghunandanan et al., 2015a). Neurosurgical interruption of CSTC circuits may also reduce OCD symptoms as well as decrease striatal volume (Rauch, 2000). Magnetic resonance spectroscopy has demonstrated alterations in glutamate metabolites in CSTC circuits (Rosenberg et al., 2004; Whiteside et al., 2006; Yucel et al., 2007), in some cases normalizing after successful treatment with an SSRI (Rosenberg et al., 2000). Evidence from the relatively few positron emission tomography (PET) ligand studies so far performed in OCD have identified abnormal binding of the serotonin transporter in cortical and subcortical areas (Reimold et al., 2007; Matsumoto et al., 2010; Hesse et al., 2011) and of the striatal postsynaptic dopamine D2 receptor (Moresco et al., 2007; Perani et al., 2008; Denys et al., 2013), which normalized after treatment with SSRI (Moresco et al., 2007).

Techniques combining gene variants and brain imaging have been used to enhance the imaging findings. Several gene variants have been found to be associated with structural and functional alteration in CSTC circuits relevant to OCRDs (reviewed in Reghunandanan et al., 2015a). For example, in patients with OCD, genetic variation in the serotonin transporter was demonstrated to be associated with reduced orbitofrontal cortex volume as measured by magnetic resonance imaging (MRI) (Atmaca et al., 2011; Hesse, 2011) and the availability of the serotonin transporter in the putamen, nucleus accumbens, and hypothalamus as measured by PET (Hesse et al., 2011).

Thus, in OCRDs, distributed network perturbation appears focused around the prefrontal cortex, caudate, putamen, and associated neurocircuitry. In OCD, convergent evidence points to deficient top-down inhibitory control in the prefrontal cortex nodes within this circuitry, coupled with the hijacking of flexible, contingency-dependent instrumental behavior in favor of excess habit generation mediated by dysfunction within the dorsal striatum (reviewed in Fineberg et al., 2014; Gillan et al., 2016a). Abnormal activation in the dorsal striatum, especially the head of the caudate nucleus and the putamen, is well replicated in the OCD literature (reviewed in Reghunandanan et al., 2015a, 2015b), implicating the cognitive fronto-striatal loop communicating with the dorsolateral prefrontal cortex driving action selection and the motor loops driving goal-directed and habitual responses (Gillan et al., 2014). This neuroanatomical model goes some way to explain the link between compulsive acts and harm-related thoughts and activities. Involvement of the putamen may be particularly relevant for the development of sensorimotor symptom such as tics. However, imaging research suggests that a wider range of CTSC circuits are involved in OCD, including systems responsible for reward processing more usually associated with addiction (Klanker et al., 2013).

Surgical disconnection of this circuitry via stereotactic capsulotomy, cingulotomy, or limbic leucotomy has been used to treat severe, intractable OCD for several decades, with some evidence of success. A double-blind, sham-controlled trial has recently produced limited evidence of the efficacy and tolerability of ventral capsulotomy using gamma radio-surgery (Lopes et al., 2014, 2015; Batistuzzo et al., 2015). Promising results from a small number of treatment studies using invasive (deep brain stimulation) or noninvasive (transcranial magnetic stimulation, transcranial direct current stimulation) methods of neuro-modulation to target either cortical (orbito-frontal cortex, presupplementary motor area [pre-SMA]) or subcortical (nucleus accumbens, subthalamic nucleus) nodes or white matter tracts within this frontal-striatal circuitry (reviewed in Senço et al., 2015) indicate new treatment possibilities for refractory obsessive-compulsive disorders. There is experimental evidence that in patients with OCD, deep brain stimulation targeted to the nucleus accumbens reduced excessive fronto-striatal connectivity within that circuit (Bourne et al., 2012). The degree of such normalization correlated with reduced severity of symptoms (Figee et al., 2013).

### Neurocognitive Models of OCRDS

Neurocognitive changes are likely to be of great value for studying the neurobiology of psychiatric disorders, as they are theoretically more directly linked to brain structure and function than are the more complex higher-level phenotypes such as compulsive symptoms (Fineberg et al., 2014). They are also more tractable to exploration across animal species (Dalley et al., 2011) and are invaluable for clinicians and patients, providing a richer understanding of the phenotype. Of the available instruments, computerized cognitive tests have several advantages over pen and paper assessment. To date, a number of tasks derived from the Cambridge Neuropsychological Test Automated Battery (CANTAB), which includes tests that are adaptable for translational work in animals and for application during brain imaging, have shown considerable utility in fractionating cognitive processes in OCRDS and in localizing neural and neurochemical substrates.

Growing evidence from human and animal research using tests such as the CANTAB suggests that the neurocognitive mechanisms mediating behavioral inhibition (motor inhibition, cognitive inflexibility) and habit formation (shift from goal-directed to habitual responding) variably contribute toward vulnerability to compulsive activity in a broad range of compulsive disorders (reviewed in Fineberg et al., 2014). Moreover, some of these deficits can be found in unaffected healthy relatives of OCD probands, suggesting they represent vulnerability or trait markers of compulsivity that also exist in nonpatient groups.

#### Motor Inhibition

Multiple tiers of evidence, ranging from functional magnetic resonance imaging (fMRI) of individuals with focal frontal lobe lesions to animal research, have demonstrated that the inhibitory control of motor acts is sub-served by a neural network linking the right inferior frontal gyrus with its subcortical (including subthalamic) connections (Rubia et al., 2003). Motor inhibition can be reliably tested using the stop-signal reaction time (SSRT) task (Aron et al., 2005). Pharmacological manipulation in rodents and humans suggests that motor response inhibition, as indexed by the SSRT, falls under the neuro-modulatory influence of the norepinephrine system (Chamberlain et al., 2006b, 2007a, 2013). In contrast, serotonin appears not to be centrally involved in this particular measure of impulsivity (Clark et al., 2005a; Chamberlain et al., 2006b; reviewed in Fineberg et al., 2014). Paradoxically, compared with the SSRIs, there is only weak evidence to suggest that drugs acting to increase norepinephrine in the synaptic cleft, such as the SNRIs venlafaxine and duloxetine (Hollander et al., 2003; Dell’Osso et al., 2008; Dougherty et al., 2015; Mowla et al., 2016), are beneficial in OCD.

In a series of experiments using the SSRT (Chamberlain et al., 2006a; Odlaug et al., 2011), evidence of significant impairment in motor inhibition, compared with healthy controls, was found in separate groups of patients with OCD, hair-pulling, and gambling disorder. However, this deficit was not seen in a study of community respondents with obsessive compulsive personality traits but without OCD (Fineberg et al., 2015), suggesting that this form of inhibitory failure represents a concomitant of compulsive motor acts. In the case of OCD, SSRT performance was also highly significantly impaired in unaffected first-degree relatives (Chamberlain et al., 2007b). In a MRI study of OCD families that included unaffected first-degree relatives (Menzies et al., 2007), reduced cortical grey matter volume, coupled with increased basal ganglia grey matter volume, was found to correlate with SSRT indices of increased motor disinhibition. This study produced some of the earliest evidence of a structural imbalance in inhibitory cortico-striatal circuitry as a neurocognitive endophenotype of motor impulsivity in OCD. A more recent fMRI study demonstrated trait-dependent compensatory hyperactivity in the pre-SMA during the performance of the SSRT in both medication-free patients with OCD and unaffected siblings vs healthy controls, representing another neurocognitive endophenotype of motor impulsivity, in this case possibly related to inefficient neural processing within the pre-SMA in those vulnerable to OCD (de Wit et al., 2012).

#### Cognitive Inflexibility

The intradimensional-extradimensional (ID-ED) shift task examines different components of attentional flexibility, including reversal learning, set formation, and inhibition, as well as shifting attention between stimulus dimensions (ED shift). Studies have demonstrated that ED shift is impaired in OCD and additionally in the unaffected first-degree relatives of OCD subjects (Chamberlain et al., 2006a, 2007b; Vaghi et al., 2016), suggesting that this aspect of cognitive inflexibility represents an endophenotype for OCD-related compulsivity. Moreover, ED shift impairment has been identified in patients with other obsessive-compulsive spectrum disorders, including obsessive-compulsive personality disorder (Fineberg et al., 2015), schizophrenia with OCD (Patel et al., 2010), and BDD (Jefferies-Sewell et al., 2016). In a study of OCD hoarders compared with compulsive hoarders without OCD, significant EDS changes versus healthy controls were found in the OCD hoarders only, suggesting that the comorbid group was associated with greater cognitive inflexibility (Morein-Zamir et al., 2014). Interestingly, another study found that hair-pulling disorder was not associated with EDS impairment, though OCD was, suggesting that cognitive inflexibility is not an essential component of repetitive acts of grooming (Chamberlain et al., 2006a).

According to a recent fMRI analysis, compared with healthy controls, patients with OCD when tested in a resting state, irrespective of treatment status, showed reduced functional connectivity in circuits linking the dorsal caudate nucleus and its anatomical cortical projections (Vaghi et al., 2017). In addition, reduced connectivity between the left dorsal caudate and the ventrolateral prefrontal cortex, an area of cortex known to be associated with EDS in healthy controls (Rogers et al., 2000), was associated with reduced OCD-related EDS performance. The reduced functional connectivity within this circuitry may account for the deficits in shifting attentional focus away from inappropriate intrusive thoughts and rituals, resulting in the perseverative behavior seen in OCD and acting as a potential biomarker of OCD.

Perseverating on a behavior that was once rewarded, but is later associated with harmful consequences, may reflect a lack of *contingency-related* cognitive flexibility. Exerting flexibility in learning and unlearning behavior based on (probabilistic) contingencies (probabilistic reversal-learning) may be particularly relevant for the development of compulsive tendencies (Fineberg et al., 2014). Contingency-related flexibility is dependent on serotonin systems (Clarke et al., 2005b) and has been linked to orbito-frontal cortex (OFC) function (Rubia et al., 2003). Reduced activation of the OFC, lateral PFC, and parietal cortex was observed using task-related fMRI during reversal learning, not only in patients with OCD but also in their unaffected, never-treated relatives (Rejminse et al., 2006; Chamberlain et al., 2008). Reversal-learning–related hypofunction, therefore, appears to be another candidate endophenotype for compulsivity that exists in people at increased genetic risk of OCD.

**Figure 3.**
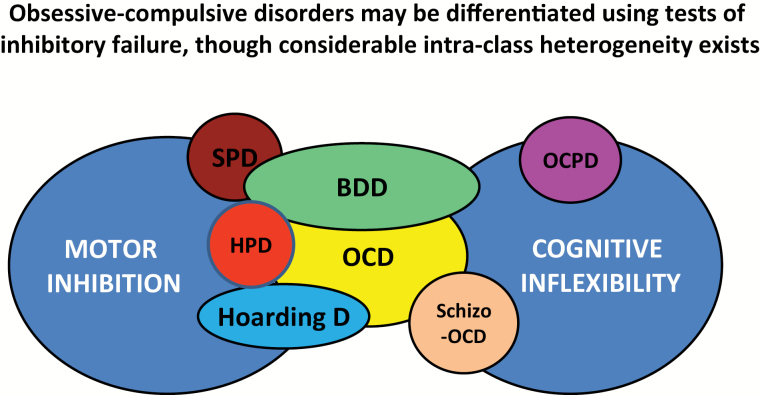
Motor Inhibition, Cognitive Inflexibility and OC Spectrum Disorders. BDD, body-dysmorphic disorder; HPD, hair-pulling disorder; OCD, obsessive-compulsive disorder; OCPD, obsessive compulsive personality disorder; schizo-OCD, schizophrenia with OCD; SPD, skin-picking disorder.

The identification of cognitive endophenotypes, such as those reflecting failures in motor inhibition and cognitive flexibility, opens new perspectives for the development of biomarkers that may be objectively quantified and used to parse compulsive disorders into more biologically homogeneous groups and that may even enable the development of personalized forms of treatment tailored to the individual (Figure 3). For example, a small-sized, randomized, placebo-controlled trial in patients with skin picking disorder found that whereas lamotrigine was not efficacious in the group as a whole, benefit was seen in a subset of patients who exhibited relatively impaired cognitive flexibility on the EDS (Grant et al., 2010). Results such as these highlight the need for randomized controlled studies of adequate power to prospectively examine the role of cognitive endophenotypes as predictors of treatment response across the full spectrum of compulsive disorders.

**Figure 4.**
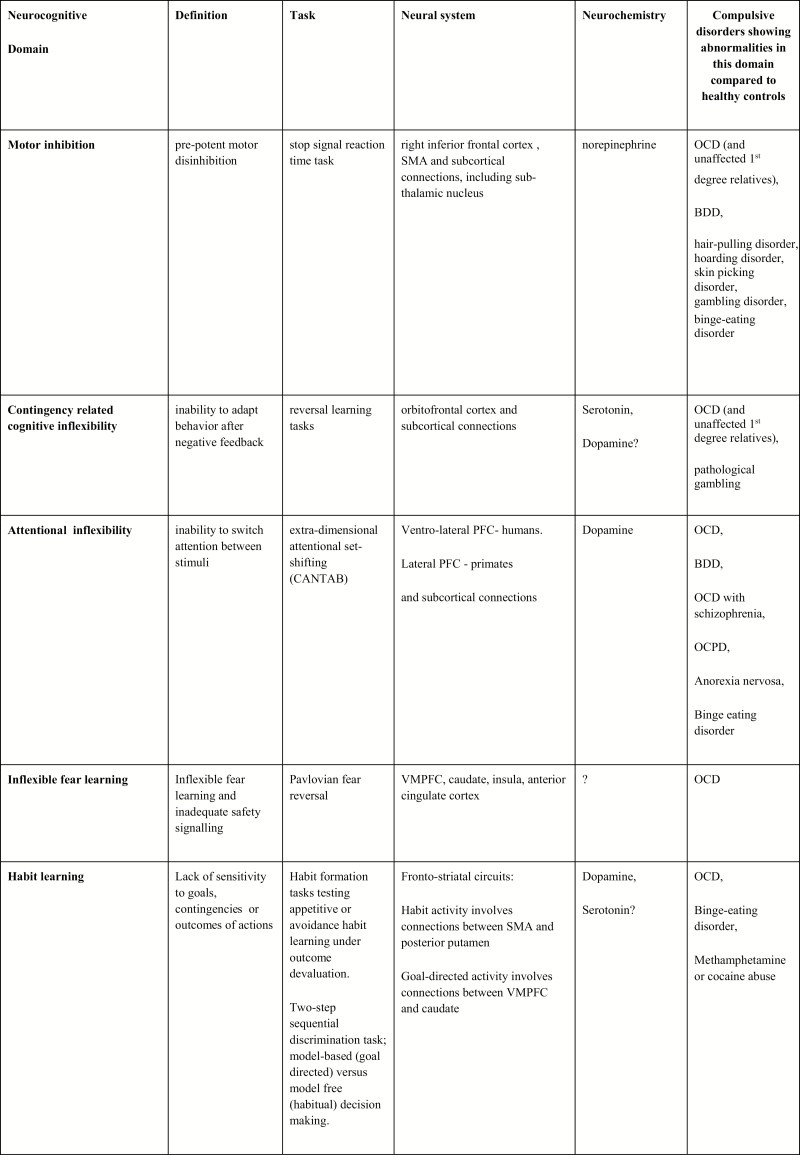
Subdividing compulsive disorders according to neurocognitive domains: task performance, neural and neurochemical correlates. Abbreviations: CANTAB, Cambridge Neuropsychological Test Automated Battery; OCPD, obsessive compulsive personality disorder; PFC, prefrontal cortex; SMA, supplementary motor area; VMPFC, ventromedial prefrontal cortex; ?, findings not assured.

#### Habit Learning

Compulsions are characterized by the persistence of activities that become disconnected from the prevailing environmental contingencies and lack an obvious relationship to the overall goal of the activity. In OCD, many patients are fully aware that their compulsive behaviors bear little to no relation to desirable outcomes, yet despite this knowledge, they continue to perform them. They often describe their compulsions as unwanted habits.

According to associative learning theories of instrumental behavior (Balleine and Dickinson, 1998; de Wit et al., 2009), actions are supported by at least 2 separate neural systems: a goal-directed system and a habitual system. When controlled by the goal-directed system, actions are purposeful inasmuch as they are flexibly performed to obtain desired goals or avoid undesired events. In contrast, habitual behaviors are considered lower order behaviors as they are performed as a routine response to specific environmental triggers and are insensitive to changes in environmental contingency (i.e., whether the action is contextually appropriate) or the outcome value of the behavior (i.e., whether the goal is actually desirable). After multiple repetitions, the habit system begins to render purposeful behavior rigid and automatic (Adams et al., 1981, 1982), allowing simple acts to be conducted without effort. Exaggerated habit formation is consistent with the ego-dystonic stimulus-driven aspects of compulsivity. Compulsivity may thus arise at least in part from a shift from goal-directed action to habit, rendering behavior insensitive to its outcome or to the prevailing environmental contingencies.

The caudate nucleus is both pivotally involved in OCD, as well as in the dynamic regulation of goal-directed contingency learning, under the prevailing influence of the ventromedial prefrontal cortex (vmPFC), which tracks the current value of outcomes. In contrast, habitual acts involve the posterior lateral putamen, where stimulus-response associations are stored (Balleine and O’doherty, 2010; Gillan et al., 2011). In a recent fMRI study of OCD patients (Banca et al., 2015), the experimental provocation of autobiographical compulsions in OCD patients was shown to reduce neural activation in brain regions implicated in goal-directed behavioral control (vmPFC, caudate nucleus) with concordant increased activation in regions implicated in habit learning (pre-SMA, putamen). This finding contrasts with previous evidence of generalized fronto-striato-limbic hyperactivation during OCD symptom evocation and provocation. This is likely due to differences in task nature and design. The cited study used a highly ecological symptom provocation paradigm, which overcame some of the limitations of previous studies, as well as, for the first time, subject-driven feedback that enabled the authors to specifically address the link between symptom provocation and compulsive urges to track the motor component of OCD. By contrast, the hyperactivation of caudate and medial prefrontal cortex found in previous studies could represent OCD-related changes in other cognitive domains such as imagery or autobiographical memory recollection, which are also processed in the medial prefrontal cortex (Lin WJ et al., 2015).

Stronger support for a shift toward habitual responding is derived from the following series of studies that investigated the extent to which patients with OCD showed a bias towards performing stimulus-response habits and away from goal-directed activities. In the first of these studies (Gillan et al., 2011), subjects were trained to respond to cues to press computer keys to win valuable points on a computer game. Next, some of the keys were devalued, that is, they were no longer linked to a valuable outcome and subjects were told not to press them when cued to do so. Yet, the OCD patients continued to habitually press in response to the cue, even after the keys had ceased to be linked to a reward.

The next study attempted to more closely model the development of compulsions as behaviors designed to avoid harmful consequences (as opposed to gain appetitive outcomes), using a shock-avoidance task (Gillan et al., 2014). Subjects were trained to lever-press in response to a computer signal to avoid a mildly painful electric shock indicated by the signal. After excessive training on the task, the electric wire was obviously disconnected and the subjects instructed not to press in response to the signal. As predicted, however, patients with OCD continued to lever-press to the devalued stimulus that explicitly no longer predicted a shock and did so significantly more than a matched healthy control group (Gillan et al., 2014). In a subsequent study, this habitual shock-avoidance behavior was directly related to fMRI evidence of hyperactivity in both the vmPFC, during the initial acquisition of the goal-directed avoidance behavior, and in the caudate, during the performance of the habitual avoidance behaviors (Gillan et al., 2015). In addition, more OCD patients than controls reported experiencing a *premonitory urge* to perform the shock-avoidance habits, the intensity of which correlated with the performance of the habits and with the strength of the fMRI caudate hyperactivity. These findings provide compelling support for the hypothesis that compulsions in OCD result from a shift from goal-directed to habitual behavioral control and are underpinned by changes in neural activity focused around the vmPFC, caudate nucleus, and the associated fronto-striatal neural circuitry.

#### Safety Signaling

The vmPFC plays a complex role in fear learning and safety-signaling and is closely involved in integrating the evaluative processing of environmental cues with flexible behavior. Abnormal vmPFC activation has been implicated in anxiety disorders (Schiller et al., 2008; Cha et al., 2014,) as well as in impaired fear retention in OCD (Milad et al., 2013). Dysfunctional processing within the vmPFC therefore represents a plausible mechanism by which explicit contingency knowledge related to safety and harm is undermined, leading to the failure to flexibly update fear responses and the persistence of rigid, habitual compulsive activity. Further studies in OCD patients are therefore under way to clarify the neuro-psychological relationship between fear and anxiety processing in the vmPFC on the one hand and cognitive flexibility in the caudate nucleus on the other. A recent neuroimaging study of Pavlovian fear reversal found that OCD patients failed to flexibly update fear responses, as measured by skin conductance changes, despite normal initial fear conditioning. This inability to update threat estimation was significantly correlated with vmPFC hyperactivation during early fear learning. The findings suggest that there is an absence of vmPFC safety signaling in OCD that potentially undermines explicit contingency knowledge and that may go some way to explain the link between cognitive inflexibility, fear, and anxiety processing in compulsive disorders such as OCD (Apergis-Schoute et al., 2017).

### Compulsive-Obsessive Disorder

In the shock avoidance studies by Gillan et al. (2014, 2015), posthoc explanations for continuing to respond to the devalued stimulus (e.g., “why did you press?”) were described as irrational threat beliefs by many of the OCD patients (e.g., “I thought I might still be shocked”). Like obsessions, these beliefs were directly contradictory to the patients’ explicit knowledge of threat and their ratings of shock expectancy. Thus, in OCD subjects faced with aversive situations, dysfunctional activation of the vmPFC and dorsal striatum may disrupt normal goal-directed behavior, leading to the generation of harm-avoidance habits that readily become compulsive or urge-driven and that may go on to create ego-dystonic, irrational fears (obsessions) with the effect of perpetuating the compulsive behavior. According to this compulsive-obsessive disorder model, the compulsive behaviors of OCD play a key role in ensuring the persistence of the obsessions.

Consistent with this model, behavioral therapy using exposure and response prevention (ERP), representing the standard psychological therapy for OCD (www. NICE.org.uk), requires patients to undergo symptom provocation via exposure to relevant stimuli or situations in order to learn to resist the urge to perform the compulsions. ERP has been found not only to produce a reduction in compulsive responding, but also concurrently causes the *urge* to respond and the associated obsessive thoughts to attenuate (Foa et al., 2005). Our data suggest that suppressing compulsions, for example using ERP, should remain a key therapeutic intervention in OCRDs and hint that habit reversal therapies (Morris et al., 2013) that are designed to break habitual associations between exposure-related cues and compulsive responses and that are currently used to treat hair-pulling disorder (Rothbart et al., 2013) may also have value in OCD, for example by augmenting the clinical response to ERP.

### Disorders of Compulsivity: A Common Bias toward Learning Habits?

A number of computational modelling techniques (e.g., the two-step sequential discrimination task; Daw et al., 2011) have been developed to infer the prevailing balance between goal-directed and habitual behavioral control by assessing a person’s decision-making tendencies (respectively, model-based versus model-free). These questionnaires and computational tasks have the added advantage of being readily disseminated on-line and therefore available to test very large numbers of subjects. In one of the earliest studies that applied this methodology to compulsivity, Voon et al. (2015a) tested a trans-diagnostic group of subjects with diagnoses involving both natural reward (binge eating disorder), artificial reward (methamphetamine/cocaine abuse), and OCD and compared them with healthy controls. The results showed a common bias across all these disorders away from model-based (goal-directed) learning. In addition, the habit formation bias was associated with lower grey matter volumes in the caudate and medial orbitofrontal cortex on structural MRI.

The findings suggested that dysfunction in a neurocomputational mechanism favoring model-free habit learning may underlie the repetitive behaviors that ultimately dominate in diverse disorders involving compulsion. In a further study (Voon et al., 2015b) that compared performance on the two-step task under conditions of reward and loss, OCD subjects compared with healthy volunteers were less goal orientated (model-based) and more habitual (model-free) to reward outcomes with a shift towards greater model-based and lower habitual choices to loss (punishment) outcomes. These results highlight the importance of motivation for learning processes in OCD and suggest that distinct clinical strategies based on reward valence may be warranted.

Most recently, Gillan et al. (2016b) applied computational modelling to investigate whether a dimensional approach could better delineate the clinical manifestations of goal-directed learning deficits using large-scale online assessment of psychiatric symptoms and neurocognitive performance in two independent general population samples. Nearly 2000 people completed the online self-report questionnaires measuring decision-making preferences as well as symptoms of various mental health conditions. As expected, people demonstrating reduced goal-directed control on the two-step task (Daw et al., 2011) also reported higher rates of compulsive symptomatology related to OCD and also eating disorder, impulse control disorder, and addiction symptoms, further demonstrating the generalizability of the deficits across multiple compulsive disorders.

By leveraging an online methodology to collect such a large dataset, Gillan et al. (2016b) were also able to deal with a key limitation of standard case-control research, the question of specificity. While the demonstration of a degree of generalizability of cognitive deficits across compulsive disorders that are similarly characterized by a loss of control over behavior, alcohol addiction, eating disorders, and impulsivity is of interest, without establishing the specificity of this deficit to this class of symptoms (and not depressive symptoms, for example), the findings are limited. By carrying out a factor analysis on their dataset, Gillan and colleagues identified that the self-report psychopathological data could be neatly fitted into three separate transdiagnostic symptom dimensions: compulsive behavior with intrusive thought, anxious-depression, and social withdrawal. Critically, they found that when the individual disorders (OCD, eating disorder, impulse control disorder, addiction) were replaced with the compulsive factor, the deficits in goal-directed control were captured even more strongly. Moreover, this association was highly specific when compared with the other noncompulsive dimensions of psychopathology. These data indicate that deficits in goal-directed control, conferring vulnerability for developing rigid habits, may have a specific role in driving the compulsive behaviors that characterize diverse disorders such as OCD, eating disorder, substance abuse, and addiction.

### Integrating Neuropsychological Models with Treatment Models

Based on these and other emerging findings, it is possible to draw inferences about the neuropsychological mechanisms underpinning the response to standard treatments in disorders such as OCD (Gillan et al., 2016a). Increased stress is known to induce a tendency to form habits (Schwabe and Wolf, 2009). It has therefore been suggested that SSRIs may act in OCD by restraining anxiety and reducing the effects of punishment, thereby helping the OCD patient to switch from habitual towards goal-directed behavior and indirectly attenuating the need to perform compulsions (Morein-Zamir et al., 2013). This effect of SSRI could also enhance the capacity to benefit from CBT with ERP (Gillan et al., 2016a). The finding from a non-randomized study that goal-directed learning under both reward and punishment conditions was enhanced in OCD patients receiving SSRI (Palminteri et al., 2012) provides some support for this hypothesis. Further support is derived from more recent findings in healthy volunteers that acute tryptophan depletion, which reduces serotonin transmission, induced a shift from goal-directed to habitual responding on a slips-of-action test and also had a deleterious effect on model-based learning (Worbe et al., 2015, 2016).

However, if the mechanism of effect of SSRI in OCD depended on anxiety reduction, benzodiazepines and other anxiolytics would also be expected to show evidence of efficacy, which they do not. Moreover, SSRIs are at their most efficacious in OCD at dosages higher than is typically recommended for anxiety disorders (Fineberg et al., 2013c; Skapinakis et al., 2016). An alternative hypothesis, therefore, proposes that SSRIs exert a therapeutic effect in OCD by bolstering goal-directed behavior through direct pharmacological actions in those areas of cortex implicated in safety signaling and goal-directed control, including the vmPFC and medial orbitofrontal cortex (El Mansari and Blier, 2006; Gillan et al., 2015; Voon et al 2015a). El Mansari and Blier (2006) reviewed the effects on 5-HT release and the adaptive changes in pre- and postsynaptic 5-HT receptor sensitivity induced by SRI treatment in rodent brain structures involved in OCD, including analogues of the OFC. The time course of increased 5-HT release and terminal 5-HT1D desensitization aligned with the course of the therapeutic response to SRI in OCD. In addition, consistent with the dose-dependent therapeutic effect of SRIs, a greater dose of SRI induced greater reuptake inhibition, which played an essential role in this phenomenon. The authors further hypothesized that the therapeutic effect of SRI-enhanced 5-HT release in the OFC is mediated by the activation of postsynaptic 5-HT2-like receptors. This cortical region is among the most consistently implicated in OCD (Whiteside et al., 2004) and shares overlapping functional connectivity abnormalities with those seen in addicted individuals at rest (Meunier et al., 2012).

Antipsychotics are used to treat stereotyped or self-injurious behavior in patients with ASD. It has been proposed that antipsychotics may also work in OCD by reducing habitual or stereotyped behavior patterns. The anti-OCD effect of antipsychotic agents, when co-administered with SSRI, has been shown to positively correlate with the drugs’ inherent dopamine D2 and D3 receptor antagonist affinities (Ducasse et al., 2014). However, whereas studies in rodents have identified a link between dopamine in the dorsal striatum and the flexible modulation of learnt behavior (Lovinger et al., 2010; Furlong et al., 2014), studies of dopamine receptor agonists and antagonists in human models of compulsive behavior have produced ambiguous findings (reviewed in Gillan et al., 2016a).

ERP for OCD involves repeated exposure to the fear-inducing stimuli that would ordinarily trigger a compulsive response, and resistance to the urge to perform the compulsion, so that the urge eventually dissipates. However, ERP usually involves weeks of practice, few people manage to drop all their compulsions, and about one-half of those with the condition are not helped at all (Reghunandanan et al., 2015b). The finding that, in OCD, patients may fail to flexibly update threat perception through faulty vmPFC safety signaling (Apergis-Schoute et al., 2017) may explain some of the difficulties that many experience in extinguishing OCD-related fears and offers exciting new treatment heuristics. The failure to recognize when a feared situation has become safe may explain why people with OCD find ERP so difficult and the treatment takes so long to work. Clinicians may therefore find these results helpful in their discussions with their patients, who could be persuaded of the importance of sticking with the therapy rather than giving up prematurely. This may even explain why co-administration of SSRI with ERP is found to be helpful. In addition, the new findings indicate the need to explore new methods of strengthening attention to safe situations during ERP to enhance fear-extinction, for example, through the use of psychopharmacological, cognitive, or neuromodulation strategies.

Another way in which the efficacy of ERP for OCD can be explained is via the systematic breaking of habitual (stimulus-response) associations between exposure-related cues and compulsive responses, achieved through repeated response-prevention exercises (Gillan et al., 2016). Brain-imaging measures of hyperactivity in the caudate nucleus of OCD patients were found to correlate both with goal-directed deficits and subjective urges to respond habitually (Gillan et al., 2014). In other studies, caudate abnormalities were found to be remediated when OCD patients respond to ERP (Baxter et al., 1992; Whiteside et al., 2012). These results suggest ERP may exert a direct effect on caudate hyperactivity. Abstinence in addiction, which results in a reduction in craving, may also work by breaking stimulus-response associations between cues and drug-taking behavior. However, both ERP and abstinence are experienced as aversive and drop-out rates are high, reflecting the need to develop more clinically acceptable ways to deliver this form of treatment. Moreover, as the habitual behaviors are strengthened with repetition (Tricomi et al., 2009), OCD and addictions become even more difficult to treat over time, emphasizing the importance of detection and intervention at the earliest stage (Fineberg et al., 2013a; Gillan et al., 2016a).

## Conclusions

The data presented serve to highlight the potential of a dimensional, biologically grounded approach to psychiatry research. They suggest that vulnerability to compulsive activity can be predicted by a spectrum of neuropsychological mechanisms, including, inter alia, impaired motor inhibition, cognitive inflexibility (attentional set-shift, reversal learning), and an imbalance in goal-directed vs habit learning. In OCD, abnormal safety signaling may undermine accurate safety learning, resulting in inflexible threat beliefs, with important implications for exposure-based therapies that rely on robust safety memories and future treatment development (Apergis-Schoute et al., 2017).

Avoidance habits, acting via disrupted goal-directed learning, represent a plausible model of chronic OCD-related compulsivity. Furthermore, emerging evidence implicates disrupted goal-directed learning in an extended group of DSM disorders characterized by compulsive behaviors and intrusive thoughts, and maps with specificity onto a trans-diagnostic compulsive symptom-dimension. Distributed network perturbation associated with these cognitive changes, affecting the prefrontal cortex (vmPFC, lateral PFC), the dorsal striatum (caudate), and the associated neuro-circuitry modulating emotional, cognitive, and motor control, has been identified in OCD. Networked studies investigating multiple disorders under the same conditions, head-to-head, are needed to determine the extent to which these changes overlap with or differentiate other disorders of compulsive behavior. Empirical evidence suggests that the psychopathology of OCRDs becomes more habitual over time. Mega-analysis in OCD suggests that illness-related structural brain changes differ in pediatric and adult cases. Longitudinal studies are now needed to explore the effect of duration of untreated disorder on the mediating neurobiology. These findings would have the potential to inform (1) the development of biomarkers to enable the detection of compulsive disorder at the earliest opportunity, crucially, in children, adolescents, and young adults, before it becomes entrenched, as well as (2) the development of new treatments with novel mechanisms of action designed to strengthen goal-directed behavior and top-down cognitive strategies for controlling urges with better efficacy and tolerability. Agreement on a standardized set of validated clinical measures of compulsivity that could be used trans-diagnostically would represent a rational next step.

## Statement of Interest

In the past several years, Dr Fineberg has received research support from Lundbeck, Glaxo-SmithKline, European College of Neuropsychopharmacology (ECNP), Servier, Cephalon, Astra Zeneca, Medical Research Council (UK), National Institute for Health Research, Wellcome Foundation, University of Hertfordshire, EU (FP7), and Shire. Dr Fineberg has received honoraria for lectures at scientific meetings from Abbott, Otsuka, Lundbeck, Servier, Astra Zeneca, Jazz pharmaceuticals, Bristol Myers Squibb, UK College of Mental Health Pharmacists, and British Association for Psychopharmacology (BAP). Dr Fineberg has received financial support to attend scientific meetings from RANZCP, Shire, Janssen, Lundbeck, Servier, Novartis, Bristol Myers Squibb, Cephalon, International College of Obsessive-Compulsive Spectrum Disorders, International Society for behavioral Addiction, CINP, IFMAD, ECNP, BAP, World Health Organization, and Royal College of Psychiatrists. Dr Fineberg has received financial royalties for publications from Oxford University Press and payment for editorial duties from Taylor and Francis. Dr Chamberlain consults for Cambridge Cognition and Shire. Dr Trevor Robbins has recently consulted for Cambridge Cognition, Mundipharma, Lundbeck, and Otsuka and has received royalties from Cambridge Cognition for CANTAB, and a research grant from Lundbeck, as well as editorial honoraria from Springer Verlag and Elsevier. Dr Barbara Sahakian consults for Cambridge Cognition, Peak, and Mundipharma and holds a grant from the Wallitt Foundation. She has in the recent past consulted for Lundbeck, Servier, and Otsuka and received grants from Peak and Janssen/JandJ.
